# Directed Evolution of an LBP/CD14 Inhibitory Peptide and Its Anti-Endotoxin Activity

**DOI:** 10.1371/journal.pone.0101406

**Published:** 2014-07-15

**Authors:** Li Fang, Zhi Xu, Guan-song Wang, Fu-yun Ji, Chun-xia Mei, Juan Liu, Guo-ming Wu

**Affiliations:** Institute of Respiratory Disease, Xinqiao Hospital, Third Military Medical University, Chongqing, China; University of Rouen, France

## Abstract

**Background:**

LPS-binding protein (LBP) and its ligand CD14 are located upstream of the signaling pathway for LPS-induced inflammation. Blocking LBP and CD14 binding might prevent LPS-induced inflammation. In previous studies, we obtained a peptide analog (MP12) for the LBP/CD14 binding site and showed that this peptide analog had anti-endotoxin activity. In this study, we used in vitro directed evolution for this peptide analog to improve its in vivo and in vitro anti-endotoxin activity.

**Methods:**

We used error-prone PCR (ep-PCR) and induced mutations in the C-terminus of LBP and attached the PCR products to T7 phages to establish a mutant phage display library. The positive clones that competed with LBP for CD14 binding was obtained by screening. We used both in vivo and in vitro experiments to compare the anti-endotoxin activities of a polypeptide designated P1 contained in a positive clone and MP12.

**Results:**

11 positive clones were obtained from among target phages. Sequencing showed that 9 positive clones had a threonine (T) to methionine (M) mutation in amino acid 287 of LBP. Compared to polypeptide MP12, polypeptide P1 significantly inhibited LPS-induced TNF-α expression and NF-κB activity in U937 cells (P<0.05). Compared to MP12, P1 significantly improved arterial oxygen pressure, an oxygenation index, and lung pathology scores in LPS-induced ARDS rats (P<0.05).

**Conclusion:**

By in vitro directed evolution of peptide analogs for the LBP/CD14 binding site, we established a new polypeptide (P1) with a threonine (T)-to-methionine (M) mutation in amino acid 287 of LBP. This polypeptide had high anti-endotoxin activity in vitro and in vivo, which suggested that amino acid 287 in the C-terminus of LBP may play an important role in LBP binding with CD14.

## Introduction

Lipopolysaccharide (LPS) is the main component of the outer wall of gram-negative bacteria and is one of the major causes of acute respiratory distress syndrome (ARDS) [1]. LPS-binding protein (LBP) and its ligand CD14 play a key role in LPS identification and transmitting LPS inflammatory signals. An LPS polymer can bind to CD14, which is located on the surfaces of macrophages, monocytes, and neutrophils, only after it is depolymerized into monomers by LBP and forms an LBP/LPS complex, that activates the LPS inflammatory signaling pathway [2]–[4]. During this process, the inflammatory effect of LPS is significantly amplified [5]. Thus, LBP/CD14 is an endotoxin sensitizing system [6].

The inflammatory effects of LPS mainly derive from the sensitizing effect of this system. An LPS/LBP/CD14 trimeric complex can induce the production of cytokines, such as tumor necrosis factor (TNF), interleukin-1, and interleukin-6, to form an inflammatory cytokine network [7], [8]. The production and release of inflammatory mediators can result in the occurrence of ARDS [9]. In addition to amplifying the LPS-mediated inflammatory signaling pathway via CD14, LBP can transport LPS to high-density and low-density lipoproteins, and thereby clear LPS from the circulation [10]–[12]. The sensitizing effect of LBP/CD14 for LPS is dependent on LBP binding to CD14. Thus, blocking the LBP binding site for CD14 might interfere with the LPS-induced inflammatory signaling pathway without affecting the scavenging activity of LBP for LPS.

In previous studies, we identified a peptide analog (MP12: FHRWPTWPLPSP) for the LBP/CD14 binding site by screening a 12-mer phage display peptide library [13]. The position of the LBP/CD14 binding site was localized to LBP amino acids 252–263. MP12 was tested at 0.1, 1.0, and 10 µg/ml. The inhibition ratio of 1.0 µg/ml and 10.0 µg/ml MP12 was evidently higher than that of 0.1 µg/ml MP12, and the inhibition ratio of 10.0 µg/ml MP12 reached 69.6%, showing concentration dependence. The LPS-induced expression of TNF-a mRNA was inhibited by MP12 (1.0, 10.0 µg/ml) in a dose-dependent manner, but it could not be inhibited by 0.1 µg/ml MP12. MP12 had anti-endotoxin effects both in vivo and in vitro. However, the working concentration of MP12 was relatively high and its anti-endotoxin effects required enhancement.

The directed evolution of proteins in vitro is an effective technique that can be used for purposeful screening by constructing numerous in vitro random mutations in genes to obtain proteins with intended functions [14], [15]. The LBP binding site for LPS is located in the N-terminus of LBP (residues 91–100) [16], [17], whereas the C-terminus of LBP binds to CD14 [18], [19]. MP12, the LBP/CD14 peptide analog obtained in our previous studies, was located in the LBP C-terminus.

Therefore, in this study, we generated a library of C-terminus mutants using error-prone PCR (ep-PCR) and phage display technology and screened for mutant peptides that could compete with LBP for CD14 binding using affinity screening and competitive inhibition experiments. We obtained a mutant peptide designated P1. This peptide fragment was located in the region of LBP amino acids 252–291. P1 inhibited the production of LPS-induced inflammatory mediators in vitro and improved the symptoms of endotoxin-induced ARDS in rats. There seemed a tendency that the anti-endotoxin activity of P1 was probably slightly stronger than that of MP12.

## Materials and Methods

### Materials

GeneMorph II random mutagenesis kit were purchased from Stratagene Company (Stratagene, USA). T7Select Phage Display System was purchased from Novagen Company (Novagen, USA). LPS and FITC-conjugated LPS were purchased from Sigma Chemicals Company (from E. coli 0111:B4, Sigma, USA). Recombinant human LBP and recombinant human CD14 and TNF-α ELISA kit were purchased from R&D Systems Company (R&D, Quantikine, USA). EMSA Kit and a Beyotime EMSA Probe Biotin Labeling Kit (NF-κB) were purchased from Thermo scientific company(Thermo, USA).All materials used in cell culture were tested by the Limulus amoebocyte lysate assay method to confirm the absence of detectable LPS, and the Limulus amoebocyte lysate was purchased from Limulus Chemicals Company of Xiamen (Xiamen,China). Phage monoclonal was donated by the Shanghai Institute of Cell Biology.

### Construction of LBP mutants and a phage peptide library

C-terminal fragments of LBP were amplified by epPCR to obtain a mutant library for the LBP C-terminus. The sequences for the restriction enzymes EcoRI and HindIII were added to the ends of the primers to allow for directional insertion into plasmids. The primer sequences for LBP were F-5′: ccggaattcCTGCGAAATGATCCAGAAATCG3′, R-5′: cccaagcttctaAACACGCTTCAGCAGAGGAAG 3′. The primer was synthesized by Shanghai Sangon. epPCR conditions were: pre-denaturation at 98°C for 5 min, 30 cycles of denaturation at 94°C for 30 sec, annealing at 57°C for 30 sec, and extension at 72°C for 30 sec, and a final extension at 72°C for 10 min. The expected length of the product was 777 bp.

PCR product ligation to pTA2 was confirmed as correct by restriction enzyme digestion and then purified and recovered using gel electrophoresis. This was attached to the EcoRI and HindIII restriction sites in pTA2 (Novagen, city, USA) by T4 DNA ligase. The ligation reaction used T7 Packaging Extracts. The ligation product was transfected into *E. coli* (BLT5403) for amplification. Capacity identification and titer determinations were done according to manufacturer instructions.

### Affinity screening of the phage display library using CD14 as substrate

We used a total of four rounds for phage screening. The affinity screening was either exactly the same as the methodology described in our previous paper [13]. For the first round of phage selection, 1.5 ml of CD14 (100 µg/ml in 0.1 M NaHCO3) was added to a 60-mm culture plate and incubated at 4°C overnight. The solution was then removed by suction and the plate was treated at room temperature with blocking solution (0.1 M NaHCO3, 5 mg/ml BSA, 0.1 g/ml streptavidin, and 0.02% w/v NaN3) for 2 h. The plate was then washed 5 times with a TBS/Tween solution (0.5%Tween 20 in 50 mM Tris–HCl, pH 7.5, 150 mM NaCl). After the last wash, 1.5 ml of phage library (4×10^10^ virions in 1.5 ml of TBS/Tween solution) was added and incubated at 4°C for 1 h. The plate was then washed 10 times with TBS/Tween, and bound phages were eluted by adding 1 ml of elution buffer (HCl adjusted to pH 2.2 with glycine, 1 mg/ml BSA, and 0.1 mg/ml phenol red) and incubated at room temperature under stirring for 8 min. The solution of eluted phages was neutralized by adding Tris–HCl (pH 9.1) and kept in the dark at 4°C until its amplification. From the second to fourth rounds of phage selection, 1.5 ml of amplified phages from previous round were submitted to the same protocol as in first round, with the exception that the final concentrations of CD14 were 10, 5, and 1 µg/ml, respectively. The bound phages in the third and fourth rounds were eluted by adding 1.5 ml of LBP (10 µg/ml, R&D Biolabs).

### ELISA for binding activity of phages

The binding activities of 48 randomly selected phage clones to CD14 were determined by ELISA. The ELISA analysis was done as described previously [13].

### Competitive inhibition of LBP binding using positive clones

Recombinant human CD14 was diluted with coating buffer to 1 µg/ml to coat the wells of a 96-well plate (100 µl/well). The plate was placed in a sealed humidified box and left at 4°C overnight. The coating solution was discarded and the blocking solution was added. Then the 96-well plate was incubated at 4°C for 2 h. After that, the blocking solution was discarded and the plate was washed 6 times with TBS/Tween. Then, 50 µl of a 1×10^10^ phage monoclonal supernatant and 50 µl of LBP (final concentration: 10 µg/ml) were added to each well. LBP and TBST at a final concentration of 10 µg/ml were added to control wells. Both plates were shaken at room temperature for 1 h. The plates were washed, an HRP-labeled goat anti-human LBP antibody (1∶500 dilution) was added, followed by shaking at room temperature for 1 h. A chemoluminescent reagent was added and phages in the wells without chemiluminescence were considered to have bound to CD14 by competing with LBP.

### DNA sequencing and polypeptide synthesis

Positive-clone phage genomic material was extracted and purified. PCR was done using a sequencing primer from the C-terminus of LBP to obtain a target fragment in the C-terminal sequence of LBP. The resultant DNA sequences were translated into peptide sequences using Chromas sequencing software. The results were compared to the rat LBP sequences obtained from GenBank. The peptide was synthesized by Shanghai Sangon company.

During polypeptide synthesis, NH2 was added to its C-terminus and N-terminal acetylation was used to enhance the stability of the polypeptide. The polypeptide obtained in this experiment was designated P1. Another polypeptide containing the MP12 sequence produced during another synthesis was designated MP12. The anti-endotoxin activities of these two polypeptides were compared.

### In vitro comparisons of anti-endotoxin activities of the two polypeptides

#### Cell culture

The human monocyte cell line U937 was cultured in accordance with the previous study [13]. Cells were treated with 15 ng/ml of phorbol myristate acetate (PMA) for 48 hours, which differentiated U937 cells into activated macrophages; adherent cells were well differentiated [20]. Cells were treated with either LPS (PBS+100 ng/ml LPs), LBP (100 ng/ml LPS+100 ng/ml LBP), P1 (10 µg/ml P1+100 ng/ml LPS+100 ng/ml LBP), or MP12 (10 µg/ml MP12+100 ng/ml LPS+100 ng/ml LBP). Blank controls were also included.

#### TNF-α production

After the above treatments, U937 cells were adjusted to 1×10^6^ cells/ml. RNA from each group of cells was extracted, purified, and then reverse-transcribed to cDNA (Promega).

The primer sequences for TNF-α were F-5′: CGAACCCCGAGTGACAAGCCTGTAGC3″, R-5′: GATCCCAAAGTAGACCTGCC3′. The expected length of the product was 474 bp. The primer sequences for β-actin were F-5′: AGTGTGACGTTGACATCCGT3″, R-5′: GACTCATCGTACTCCTGCTT3′. The expected length of the product was 244 bp. The primer was synthesized by Shanghai Sangon. RT-PCR conditions were: pre-denaturation at 94°C for 3 min, 30 cycles of denaturation at 94°C for 30 sec, annealing at 57°C for 30 sec, and extension at 72°C for 40 sec, and a final extension at 72°C for 10 min(for both TNF-α andβ-actin). Electrophoretograms were acquired with a gel imaging system and semi-quantitative analysis used Quantity One image analysis software. The optical density ratio for TNF-α/β-actin was determined.

The number of U937 cells was adjusted to 1×10^6^ cells/ml with culture medium and treated or not treated for 4 h as previously described [13]. The cell suspension was transferred to the vials and centrifuged at 1,500 rpm for 5 min. The supernatant were removed and the concentrations of TNF-α in the supernatant were evaluated with a human TNF-α ELISA kit.

#### EMSA assay for NF-κB activity

We used a Thermo Scientific LightShift Chemiluminescent EMSA Kit and a Beyotime EMSA Probe Biotin Labeling Kit (NF-κB) to determine NF-κB activity. Probe sequences: 5′-AGT TGA GGG GAC TTT CCC AGG C-3′, 3′-TCA ACT CCC CTG AAA GGG TCC G-5′. According to the kit manual, Biotin end-labeled DNA and protein extracts (5 µg) were co-incubated at room temperature for 20 min. These were separated using non-denaturing 5% (w/v) polyacrylamide gel electrophoresis(180 V,45 min). Subsequently, DNA was quickly transferred to a nylon membrane using a positive electrode(380 mA,30 min), treated by UV cross-linking, and detected by HRP-labeled streptavidin using standard procedures. TotalLab Quant Software was used to obtain integrated optical density (IOD) values.

### Effects of P1 and MP12 polypeptides on LPS-induced ARDS in rats

Animal experiments were approved by the Animal Care and Use Committee of the Third Military Medical University. Wistar rats (10–12 weeks of age, 178±11 g) were purchased from the Experimental Animal Center of the Third Military Medical University. Prior to experiments, rats were maintained in the animal house for one week to adapt to their environment. Rats were divided into 4 groups (10 rats/group) which include control group, LPS group, LPS+MP12 group and LPS+P1 group. For the LPS group, LPS was diluted with 500 µl of saline and injected through the cervical vein (5 mg/kg body weight). For the P1 group, P1 (5 mg/kg body weight) was injected just after LPS (5 mg/kg body weight) [21], [22] injection. For the MP12 group, MP12 (5 mg/kg body weight) was injected just after LPS injection. For the control group, saline was used in place of the materials noted above. Then rats were maintained in the animal house for observation. At 2 hours after injection, Arterial partial pressure of oxygen(PaO2) and Oxygenation index (PaO2/FiO2) was done using arterial blood withdrawn from the carotid artery using a sterile heparin-treated syringe. We had taken 1% pentobarbital sodium to 40 mg/Kg dose of anesthesia to the rats before drawing carotid arterial blood. The rats were put to death by using excess anesthesia at 72 hours after injection and some parts of the lung tissue were harvested. These tissues were fixed in 10% formalin for histopathology. All invasive operations (including LPS or saline injection and extraction of arterial blood gas analysis, etc.) were performed in the presence of anesthesia.

We took care of rats(20 rats/group) during experiments and ensured water, food, a clean and quiet environment were provided and maintained a strict observation of the animals behaviour and health. It was thought that rats were close to death when the rats appeared the following clinical features (faint breathing, amyasthenia, or tremor, lack of independent reaction to the outside world, cyanosis or coma), then the human endpoints would be operated by injecting with excessive anesthesia. The number of such rats was recorded as ‘rat mortality’.

### Pathological evaluation and semi-quantitative analysis of rat lung tissues

Using routine methods, rat lung tissue was fixed,paraffin embedded, and then cut into 5 µm slices. H&E staining was used for these sections. The pathology of the lung tissues were assessed by light microscopy. Scoring of lung tissues included pulmonary edema, neutrophil infiltration, alveolar hemorrhage, hyaline membrane formation, and lung atelectasis. Scores of “0, 1, 2, and 3” were recorded that corresponded to “none, mild, moderate, and severe” changes. The individual scores for each parameter were compared (n = 20 for each group).

### Statistical analysis

SPSS 11.0 was used for statistical analysis. Results are given as means ± standard deviations. Multiple group comparisons were made by a non-parametric test and Kruskal-Wallis test by a Dunns post-test was used to compare the variances between groups. P<0.05 was considered statistically significant and P<0.01 was considered highly significant.

## Results

### Establishing the LBP mutant phage peptide library

**Figure 1:**
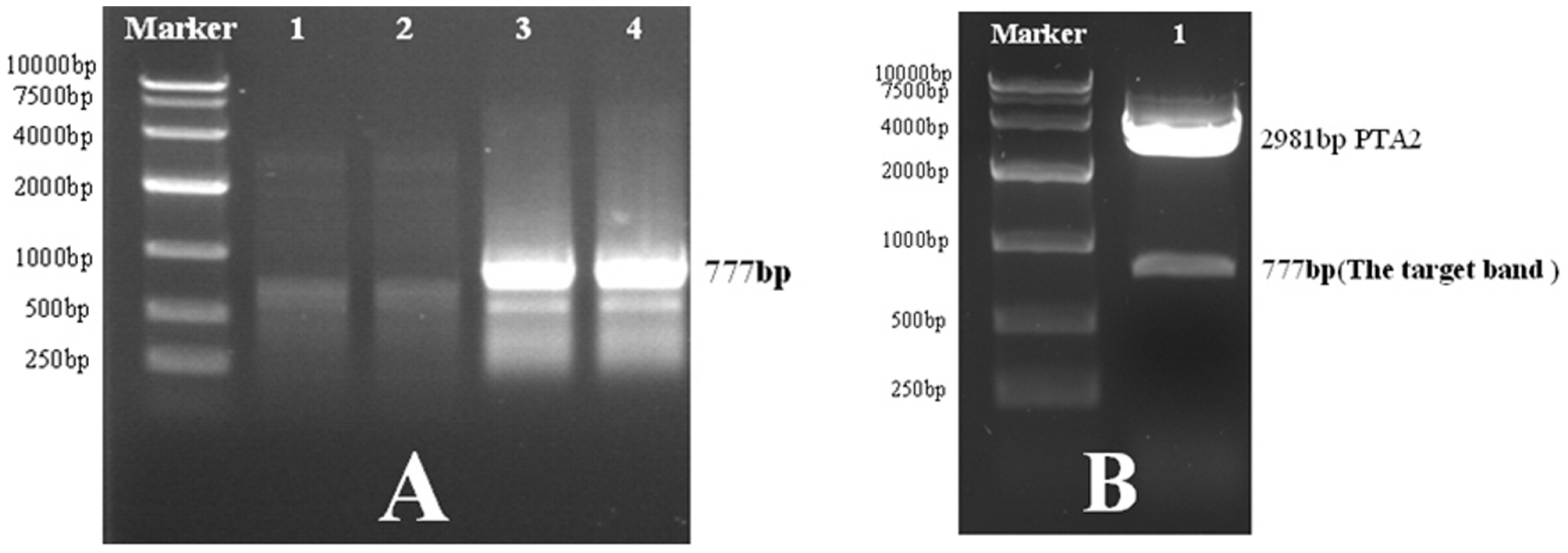
Establishing the LBP mutant phase library. A. The first and second lanes are the PCR products obtained after annealing at 59°C. The third and fourth lanes are the PCR products obtained after annealing at 57°C and these were collected. B. Restriction enzyme digestion after ligation of the PCR products with PTA2. The target band appeared in the 777-bp fragment (lane 1).

Agarose gel electrophoresis results for epPCR products appeared as a 777-bp fragment. An epPCR product was ligated to a PTA2 carrier. Four clones were randomly selected and restriction enzyme digestion was successful (Figure 1A, B). These were then assembled with T7 phages and an LBP mutant phage peptide library was produced. Several single colonies were selected for sequencing to determine that a target gene and its carrier were successfully attached. The library capacity was 6.4×10^8^ and the titer was 4×10^12^ pfu/ml.

### Screening positive phages with affinity for CD14

Affinity screening with CD14 as the target molecule was done for the LBP mutant phage peptide library. After four rounds of screening, effective enrichment of target phage clones was achieved.

### Screening for positive phage binding to rhCD14

**Figure 2:**
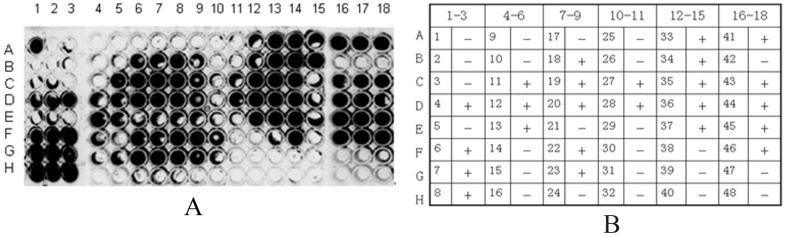
Chemiluminescence assay for phage binding activity with CD14. A. The luminescent particles were single clones with binding activity (3 independent experiments done in duplicate). A1–3, and B1–3 to H16–18 (3 independent experiments done in duplicate) were for samples 1 to 48, respectively. B. Chemiluminescence plate in (A). Corresponding single clone numbers and binding activity status. “+” indicates binding activity, “−” indicates no binding activity.

After four rounds of phage screening, 48 single clones were randomly selected. Chemoluminescent detection showed that 24 phage clones (No. 4, 6, 7, 8, 11, 12, 13, 18, 19, 20, 22, 23, 27, 28, 33, 34, 35, 36, 37, 41, 43, 44, 45, 46) had relatively high binding to CD14. These 24 clones were used in competitive inhibition tests against LBP (Figure 2).

### Competitive binding for CD14 between LBP and phage clones

**Figure 3:**
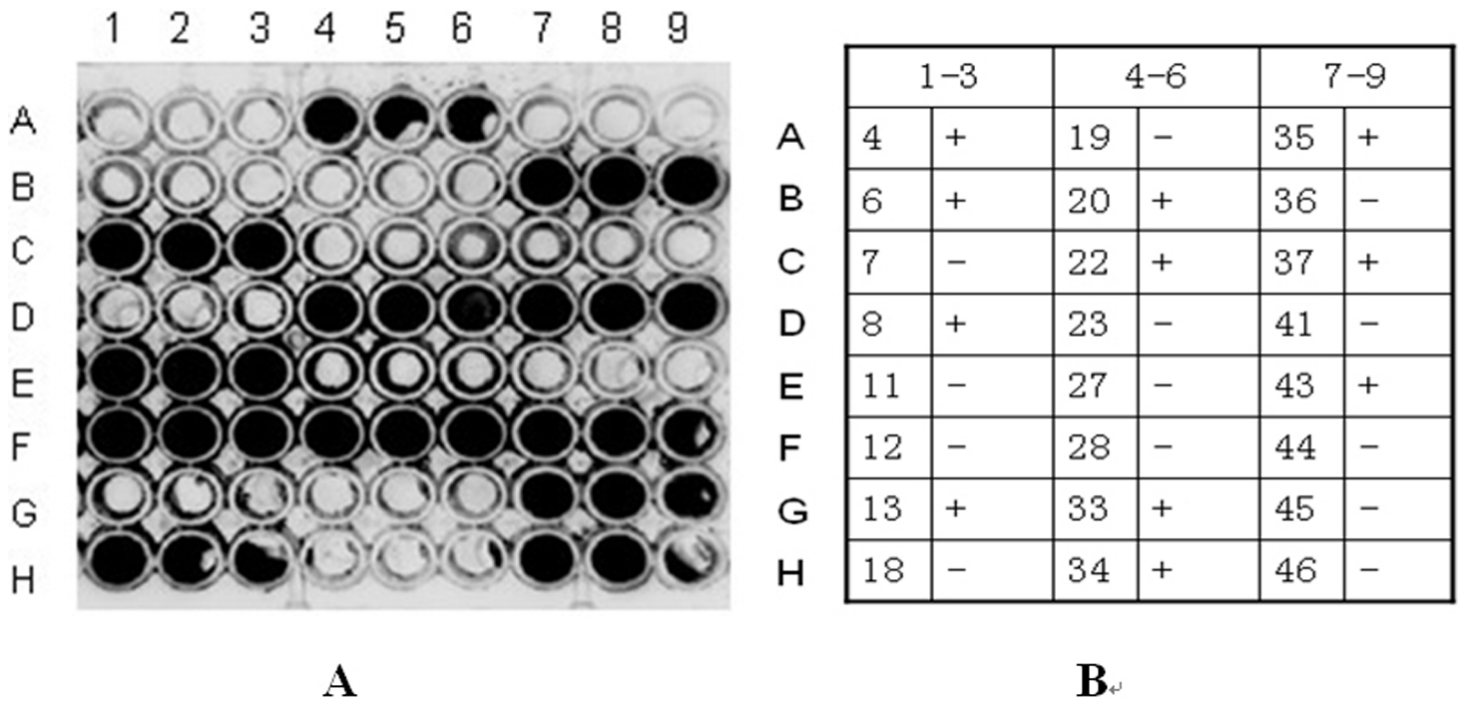
Chemiluminescent detection of competitive inhibition of LBP binding to CD14 by phage clones. A. Non-luminescent particles indicated clones with competitive binding capacity. B. Number of single clones corresponding to the chemiluminescence plate labels and their competitive inhibition status. “+” indicates competition with LBP, “−” indicates no competition.

The 24 positive bound to rhCD14 by competing with LBP. These results showed that 11 of 24 clones(No. 4, 6, 8, 13, 20, 22, 33, 34, 35, 37, 43) could bind to rhCD14 by competing with LBP.Non-luminescent particles indicated clones with competitive binding capacity (3 independent experiments done in duplicate). (figure 3).

### DNA sequencing

DNA sequencing was done for the 11 clones that competed with LBP for CD14 binding. and the resultant DNA sequences translated into peptide sequences using Chromas sequencing software. The results were compared to the rat LBP sequences obtained from GenBank. These sequencing results showed that threonine (T) to methionine (M) mutations had occurred at amino acid 287 of LBP in 9 clones (No. 4, 6, 13, 20, 33, 34, 35, and 39) (Table 1).

**Table 1:** Sequences of 11 clones obtained from screening.

Clones number	Sequences of 11 clones
No. 4 clone	DYSLVEAPRATAQMLEVMFKGEIFHRNHRSPVTLLAAVMSLPEEHNKMVYFAISDYVFNMASLVYHEEGYLNFSITDDMIPPDSNIRLTTKSFRPFVPRLARLYPNMNLELQGSVPSAPLLNFSPGNLSVDPYMEIDAFVLLPSSSKEPVFRLSVATNVSATLTFNTSKITGFLKPGKVKVELKESKVGLFNAELLEALLNYYILNTFYPKFNDKLAEGFPL
No. 6 clone	CEMIPEIGVLRSTALSPKLCQLQQRLTVFADIDYSLVEAPRATAQMLEVMFKGEIFHRNHRSPVTLLAAVMSLPEEHNKMVYFAISDYVFNMASLVYHEEGYLNFSITDDMIPPDSNIRLTTKSFRPFVPRLARLYPNMNLELQGSVPSAPLLNFSPGNLSVDPYMEIDAFVLLPSSSKEPVFRLSVATNVSATLTFNTSKITGFLKPGKVKVELKESKVGLFNAELLEALLNYYILNTFYPKFNDKLAEGFPLPLLKRV
No. 8 clone	TSPSQINMKNA*L*YRGVHQVQKDPSSLG*PGSTPT*TWKNKDQCPRLRPELQPWESVCGPLYGDRCLSAPAQLQQGACLPAQCGQ*CVRHLNLQYQQDHWVPEARKGKSGSERIKKVGLFNAELLEALLFFMTSLHLLPQVPPLIYFRRLPPSLRGPS
No. 13 clone	AKMIQKSVSSDLQPYLQTLSVTTEIDSFADIDYSLVEAPRATAQMLEVMFKGEIFHRNHRSPVTLLAAVMSLPEEHNKMVYFAISDYVFNMASLVYHEEGYLNFSITDDMIPPDSNIRLTTKSFRPFVPRLARLYPNMNLELQGSVPSAPLLNFSPGNLSVDPYMEIDAFVLLPSSSKEPVFRLSVATNVSATLTFNTSKITGFLKPGKVKVELKESKVGLFNAELLEALLNYYILNTFYPKFNDKLAEGFPLPLLKRV
No. 20 clone	FGQQPRCWEVMFKGVKFFHRNPPVLQLPSFAAVMSLS*GTQQNGLLCHLGIMSFNMASLVYHEEGYLNFSITDDMIPPDSNIRLTTKSFRPFVPRLARLYPNMNLELQGSVPYAPLLNFSPGNLSVDPYMEIDAFVLLPSSSKEPVFRVSVATNVSATLTFNTSKITGFLKPGKVKVELKESKVGLFNAELLEALLIYYILNTFYPKFIFFFPKASPFLC
No. 22 clone	WEMIQKSVSSDLQLYLQTLSVTTEIDSFADIDYSLVEAPRATAQMLEVMFKGEIFHRNHRSPVTLLAAVMSLPEEHNKMVYFAISDYVFNMASLVYHEEGYLNFSITDDMIPPDSNIRLTTKSFRPFVPRLARLYPNMNLELQGSVPSAPLLNFSPGNLSVDPYMEIDAFVLLPSSSKEPVFRLSVATNVSATLTFNTSKITGFLKPGKVKVELKESKVGLFNAELLEALLNYYILNTFYPKFNDKLAEGFPLPLLKRV
No. 33 clone	SRNQVSSDLQPYLQNSVSYNRD*QFRRH*L*LSGSPSGQQPRCWR*CLRGEIFHRNHRSPVTLLAAVMSLPEEHNKMVYFAISDYVFNMASLVYHEEGYLNFSITDDMIPPDSNIRLTTKSFRPFVPRLARLYPNMNLELQGSVPSAPLLNFSPGNLSVDPYMEIDAFVLLPSSSKEPVFRLSVATNVSATLTFNTSKITGFLKPGKVKVELKESKVGLFNAELLEALLNYYILNTFYPKFNDKLAEGFPLPLLKRV
No. 34 clone	SQRSVSSDLQPYLQTLSVTNRD*QFSPTLIISLVGSPLGQQPRCWR*CLRVKIFHRNHRSPVTLLAAVMSLPEEHNKMVYFAISDYVFNMASLVYHEEGYLNFSITDDMIPPDSNIRLTTKSFRPFVPRLARLYPNMNLELQGSVPSAPLLNFSPGNLSVDPYMEIDAFVLLPSSSKEPVFRLSVATNVSATLTFNTSKITGFLKPGKVKVELKESKVGLFNAELLEALLNYYILNTFYPKFNDKLAEGFPLPLLKRV
No. 35 clone	TLSVTTEIDSFADIDYSLVEAPRATAQMLEVMFKGEIFHRNHRSPVTLLAAVMSLPEEHNKMVYFAISDYVFNMASLVYHEEGYLNFSITDDMIPPDSNIRLTTKSFRPFVPRLARLYPNMNLELQGSVPSAPLLNFSPGNLSVDPYMEIDAFVLLPSSSKEPVFRLSVATNVSATLTFNTSKITGFLKPGKVKVELKESKVGLFNAELLEALLNYYILNTFYPKFNDKLAEGFPLPLLKRV
No. 37 clone	GGK*VTRVRPQASKHASAEEGGSLRLNIIELGVEGVKAAIVEERFEQLCLE*SNFGFFQFHFYLSWLQDPSDLAGTERQGGGHISGPTALPPQAPWWSSPRPIKAPILLLGVCPQIRMAETKAPRKGN*V
No. 43 clone	RNDPEIVSSDLQPYLQTLSVTTEIDSFADIDYSLVEAPRATAQMLEVMFKGEIFHRNHRSPVTLLAAVMSLPEEHNKMVYFAISDYVFNMASLVYHEEGYLNFSITDDMIPPDSNIRLTTKSFRPFVPRLARLYPNMNLELQGSVPSAPLLNFSPGNLSVDPYMEIDAFVLLPSSSKEPVFRLSVATNVSATLTFNTSKITGFLKPGKVKVELKESKVGLFNAELLEALLNYYILNTFYPKFNDKLAEGFPLPLLKRV

Note:

1. Sequences of No. 198-456 amino acids of LBP: CEMIQKSVSSDLQPYLQTLPVTTEIDSFADIDYSLVEAPRATAQMLEVMFKGEI**FHRNHRSPVTLL**AAVMSLPEEHNKMVYFAISDYVFNTASLVYHEEGYLNFSITDDMIPPDSNIRLTTKSFRPFVPRLARLYPNMNLELQGSVPSAPLLNFSPGNLSVDPYMEIDAFVLLPSSSKEPVFRLSVATNVSATLTFNTSKITGFLKPGKVKVELKESKVGLFNAELLEALLNYYILNTFYPKFNDKLAEGFPLPLPRRI.

2. The highest mutation frequency was observed in amino acid 287 (T to M mutation) at a rate of 81.8%, followed by a P to S mutation in amino acid 217 at a rate of 36.4%. The remaining rate was 9.1%.

Based on these results, we synthesized an artificial polypeptide containing amino acids 252–291 of LBP, which included the mutation site noted above; this was designated P1. Its sequence was: FHRNHRSPVTLLAAVMSLPEEHNKMVYFAISDYVFNMASL We also synthesized another polypeptide, designated MP12, which contained amino acids 252–291 of LBP. The only difference was that the sequence of amino acids 252–263 was substituted with FHRWPTWPLPSP, which was obtained in our previous experiments. Its polypeptide sequence was: FHRWPTWPLPSPAAVMSLPEEHNKMVYFAISDYVFNTASL.

### Effects of polypeptides P1 and MP12 on LPS-induced TNF-α mRNA expression

**Figure 4:**
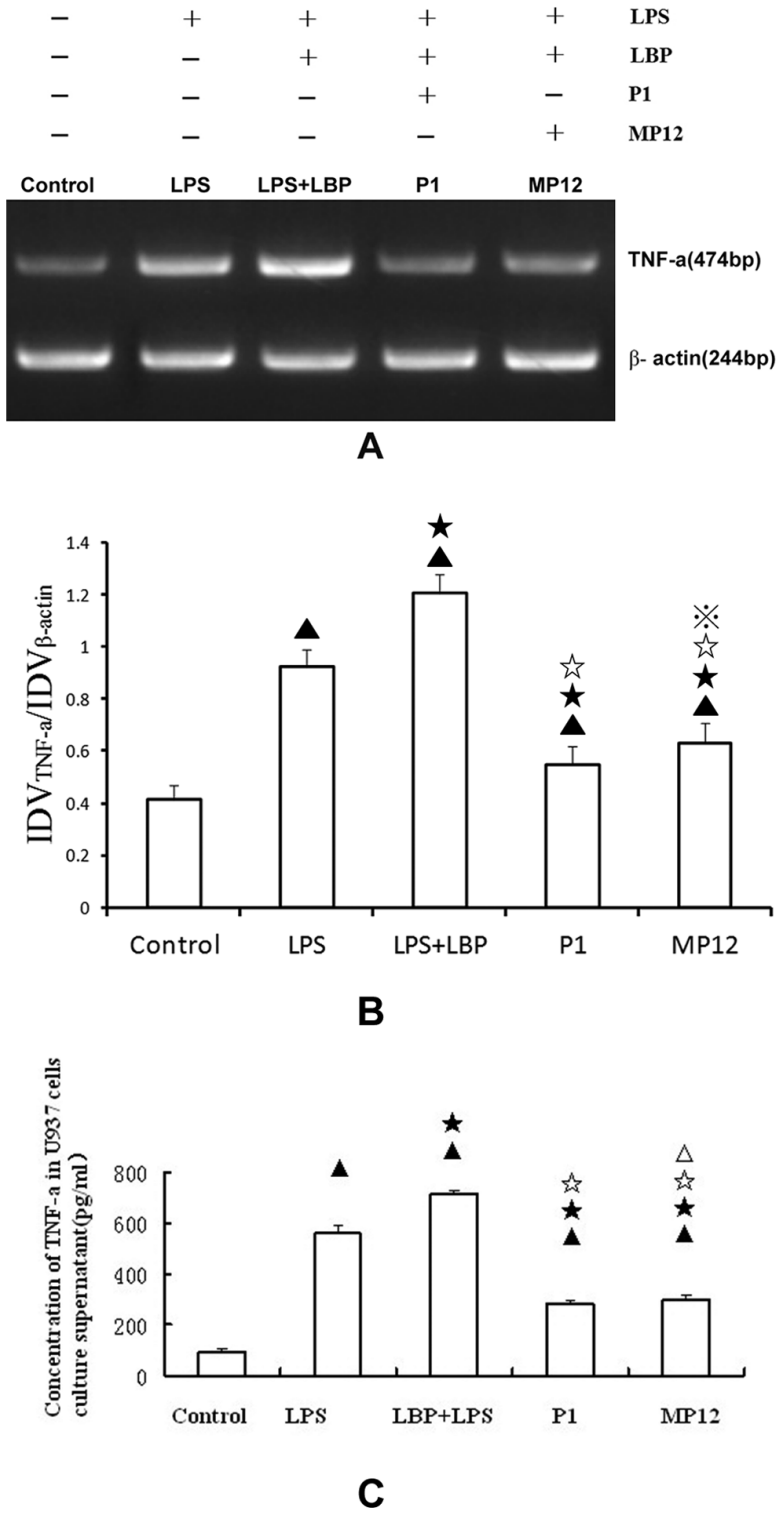
Effects of polypeptides P1 and MP12 on LPS-induced TNF-α mRNA expression and secretion by U937 cells. A. Agarose gel electrophoresis results for TNF-α mRNA expression by RT-PCR. B. The optical density ratio for target gene/β-actin was determined. Results are means ± standard deviations. C. Cell culture supernatant was used to detect the concentration of TNF-α(with ELISA) ▴ compared with the control: P<0.01; ★ compared with LPS treatment: P<0.01; ☆ compared with LPS+LBP treatment: P<0.01; 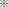 compared with P1 treatment: P<0.05; ▵ compared with P1 treatment:P<0.01.

After agarose gel electrophoresis, the integrated density value (IDV) of each band was measured using an EASY image analysis system(n = 15). Mature U937 cells had trace amounts of TNF-α mRNA expression(0.417±0.016 for the control group). LPS treatment significantly increased their TNF-α mRNA expression(0.921±0.019 for the LPS group) and LBP significantly enhanced that(1.205±0.024 for the LPS+LBP group) (LPS+LBP treatment versus LPS treatment; P<0.01). Polypeptides P1(0.549±0.023 for the P1 group) and MP12(0.631±0.025 for the MP12 group) significantly reduced LBP/LPS-induced TNF-α mRNA expression (P1 treatment and MP12 treatment versus LPS+LBP treatment; P<0.01). Polypeptide P1 inhibition of LBP/LPS-induced TNF-α mRNA expression was greater than that by MP12 (P1 treatment versus MP12 treatment; P<0.05). Quantitative assessment of TNF-α in cell culture supernatants measured by ELISA indicated that LPS treatment significantly increased the secretion of TNF-α(560.52±28.63 pg/ml for the LPS group versus 94.19±7.02 pg/ml for control group,P<0.01,n = 6) and LBP significantly enhanced that(715.65±11.94 pg/ml for the LPS+LBP group) (LPS+LBP treatment versus LPS treatment; P<0.01). Polypeptides P1(286.38±7.42 pg/ml for the P1 group) and MP12(301.29±12.17 pg/ml for the MP12 group) significantly reduced LBP/LPS-induced the secretion of TNF-α(P1 treatment and MP12 treatment versus LPS+LBP treatment; P<0.01). Polypeptide P1 inhibition of LBP/LPS-induced the secretion of TNF-α was significantly greater than that by MP12 (P1 treatment versus MP12 treatment; P<0.01) (Figure 4).

### Effects of polypeptides P1 and MP12 on LPS-induced NF-κB activity

**Figure 5:**
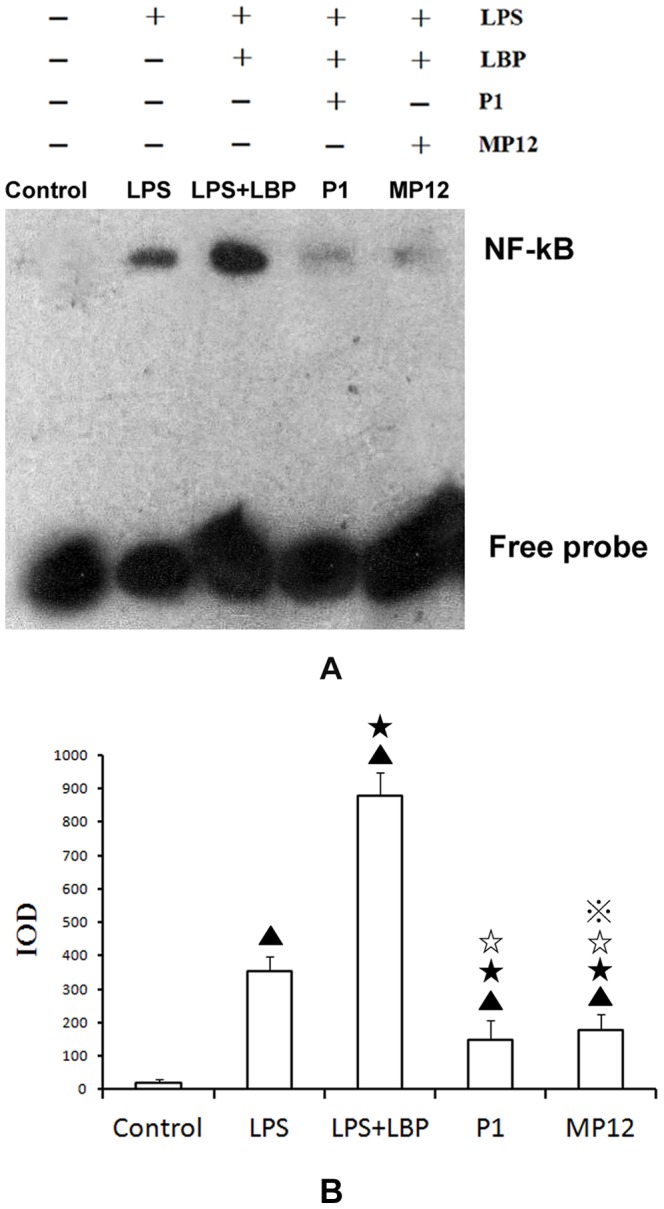
Effects of P1 and MP12 on NF-κB activity in U937 cells. A. NF-κB binding was significantly increased by LPS treatment. After treatment with P1 or MP12, NF-κB binding decreased; a more significant decrease was found with P1 treatment compared to MP12 treatment. B. IOD values were obtained using TotalLab Quant software. ▴ compared with the control: P<0.01; ★ compared with LPS treatment: P<0.01; ☆compared with LPS+LBP group: P<0.01; 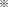 compared with P1 group: P<0.05.

As described in the methods section, EMSA was used to assess NF-κB binding in U937 cells after different treatments, then integrated optical density (IOD) values were obtain by TotalLab Quant Software(n = 6). Mature U937 cells had minimal NF-κB binding(20.67±6.17 for control group). LPS treatment significantly increased NF-κB binding (352.87±14.78 for the LPS group) and LBP significantly enhanced that(868.37±43.91 for the LPS+LBP group) (LPS+LBP treatment versus LPS treatment; P<0.01). Polypeptides P1 and MP12 significantly decreased LBP/LPS-induced NF-κB binding (P1 treatment and MP12 treatment versus LPS+LBP treatment; P<0.01). Polypeptide P1(147.68±16.79 for P1 group) inhibition of LBP/LPS-induced NF-κB binding was significantly greater than that by MP12(176.44±15.86 for MP12 group) (the P1 group versus the MP12 group, P<0.05) (Figure 5).

### Rat mortality in each group

After injury induced by LPS, 15 of 20 rats in the LPS group had died(died in 8 hours,10 hours,12 hours,14 hours,16 hours,18 hours,24 hours,32 hours and 38 hours after injection, respectively). By comparison, 5 of 20 rats in the P1 group died(died in 50 hours,52 hours,54 hours,58 hours after injection, respectively), 8 of 20 rats in the MP12 group died (died in 34 hours,36 hours,38 hours,42 hours and 56 hours after injection, respectively), and no rats died in the normal group.

### Effects of polypeptides P1 and MP12 on arterial oxygen partial pressure and oxygenation index of rats with LPS-induced acute respiratory distress syndrome

**Figure 6:**
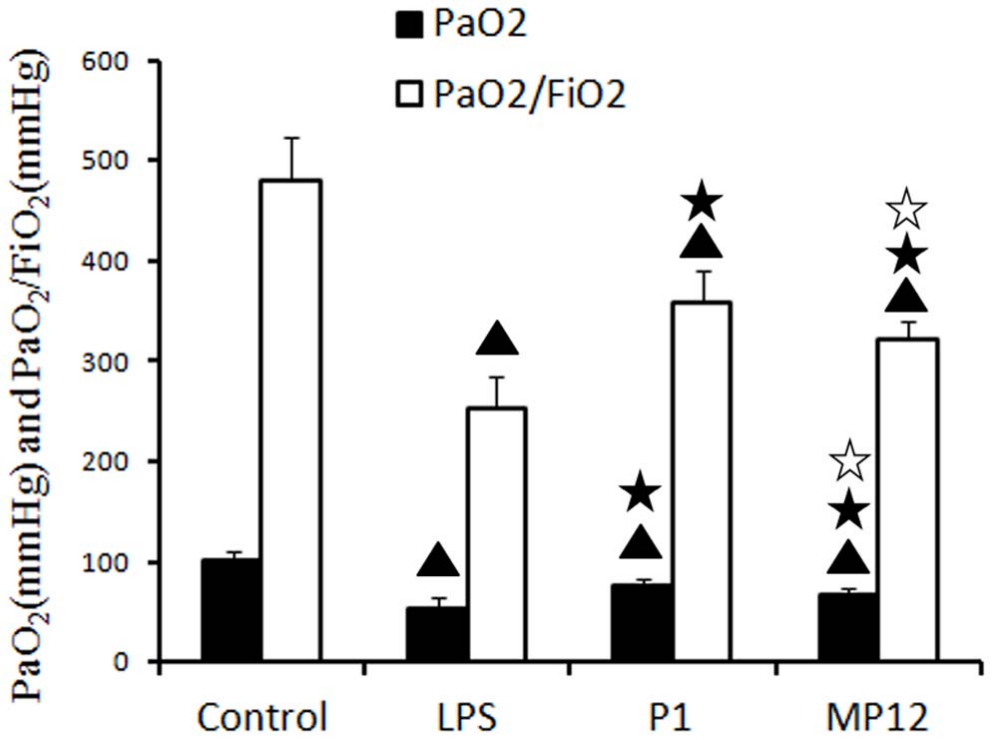
Effects of P1 and MP12 on LPS rat arterial blood gas analysis. PaO2 and PaO2/FiO2 decreased significantly in the LPS group compared to the normal group; with LPS, these were <60 mmHg and <300 mmHg, respectively. After P1 and MP12 treatments, both PaO2 and PaO2/FiO2 improved; these were >60 mmHg and >300 mmHg, respectively. The outcome was better in the P1 group compared to the MP12 group. ▴ compared with the control group: P<0.01, ★ compared with the LPS group: P<0.01; ☆ compared with the P1 group: P<0.05.

After LPS injection, arterial oxygen partial pressure (PaO_2_) and an oxygenation index (PaO_2_/FiO_2_) decreased significantly (PaO_2_53.29±6.26 mmHg, PaO_2_/FiO_2_ 253.74±29.83, n = 7) compared to normal (PaO_2_100.85±9.09 mmHg, PaO_2_/FiO_2_ 480.24±43.29, n = 6), untreated rats (P<0.01). With LPS injection, PaO_2_ and PaO_2_/FiO_2_ were <60 mmHg and <300 mmHg, respectively. Both polypeptides P1 and MP12 improved the PaO2 and PaO2/FiO2 values of rats with ARDS; these improvements were more significant in the P1 group (PaO_2_75.27±6.68 mmHg, PaO_2_/FiO_2_ 358.41±31.8, n = 6) compared to the MP12 group (PaO_2_67.42±1.89 mmHg, PaO_2_/FiO_2_ 321.03±9.01, n = 6) (P<0.05). The mean PaO_2_ and PaO_2_/FiO_2_ values were >60 mmHg and >300 mmHg, respectively, in the P1 group and the MP12 group (Figure 6).

### Pathological evaluations and semi-quantitative scoring of rat lung tissues after LPS-induced ARDS

**Figure 7:**
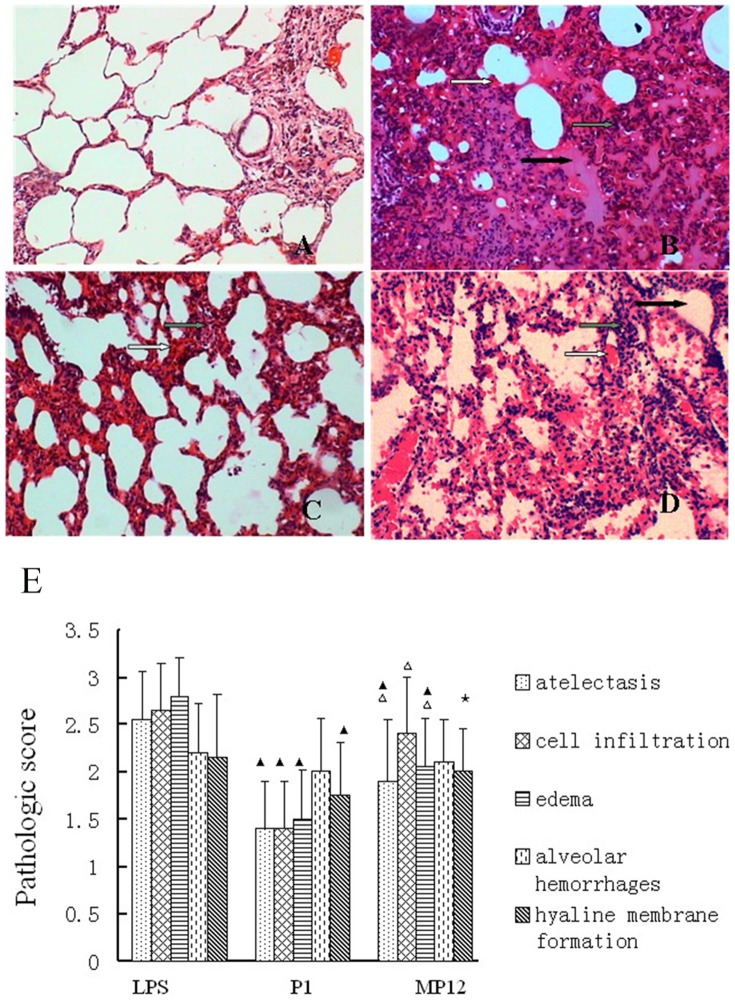
Pathological evaluations and scoring of rat lung tissues. I. Rat lung tissue under a light microscope. A. Rat lung tissue had a relatively complete structure in the normal control group. B. Rat lung tissue in rats with LPS-induced injury. C. Rat lung tissue in P1 group. D. Rat lung tissue in MP12 group. Edema: black arrow; congestion: white arrow; inflammatory cell infiltration: gray arrow. II. Pathological scoring for lung tissue:(the scores of control group is zero) ▴compared with the LPS group: P<0.01; ▵ compared with the P1 group: P<0.01;★compared with the P1 group: P<0.05.

Rat lung tissue had a relatively complete structure in the normal control group (Figure 7 IA). Alveolar spaces were clear and there were no pathological changes, such as edema or inflammation. In rats with LPS-induced injury, the pulmonary interstitium became significantly wide with massive inflammatory cell infiltration, effusion was noted in the alveolar space, and local atelectasis could be observed. (Figure 7 IB). After treatments with P1 and MP12, pulmonary edema and pulmonary hemorrhage decreased and neutrophil accumulation was significantly reduced (Figure 7 IC, ID). After intravenous LPS injection, the semi-quantitative scores for pulmonary pathology were significantly higher than those of normal rats (P<0.01). The P1 group and the MP12 group had scores lower than those of the LPS group (P<0.01). Moreover, the score for the P1 group was lower than that for the MP12 group (Figure 7 II).

## Discussion

LBP/CD14 has a strong sensitizing effect on LPS-induced inflammation [23]–[25]. It was reported that a very small amount of LPS could induce the release of inflammatory factors in the presence of LBP/CD14, whereas interfering with LBP/CD14 could significantly reduce LPS-induced inflammation. Thus, interfering with LBP/CD14 interactions has been considered for treating endotoxin-induced diseases, such as ARDS and sepsis [26], [27].

It has also been found that LBP can bind to LPS via the N-terminus of this peptide. This structural domain is located in its amino acid residues 91–100 [28]. However, the C-terminus of the LBP peptide chain may contain a structural domain that can bind to CD14. When the C-terminus of the peptide chain is absent, a small amount of LPS will not induce cells to produce inflammatory cytokines. The C-terminus of the LBP peptide chain comprises amino acid residues 198–456. The structural domain for binding to CD14 has not yet been accurately localized [29]. Thus, the structure and functions of the C-terminus in the LBP chain and the LBP site for binding to CD14 remain to be studied. Blocking this site with a synthesized peptide analog should interfere with the sensitizing effect of LBP/CD14 on LPS without affecting LPS clearance by LBP.

In our previous studies [13], we successfully screened a phage-displayed 12-mer peptide library and found a peptide analog, MP12, which was located in amino acids 252–263 in the C-terminus of LBP. This peptide could block the binding site of LBP/CD and had anti-endotoxin activity both in vivo and in vitro. However, the working concentration of this peptide analog was relatively high and its anti-endotoxin activity required further improvement.

Recently, directed evolution of proteins in vitro was successfully used to modify the expected functions of proteins [30], [31]. Thus, we attempted to establish a mutant library for the C-terminus of LBP (the site for LBP binding to CD14) using epPCR and phage display technology. By affinity screening and competitive inhibition tests, we obtained a mutant peptide that could block the LBP site for binding to CD14. The anti-endotoxin activities of this mutant peptide and the previously obtained peptide analog were compared.

In the current study, the mutation frequency of DNA fragments in the C-terminus of LBP was 0.39%–1.55%. This was in accordance with the mutation rate at a level of 2–3 nucleotides in each sequence or an amino acid residue mutation, which can be used to generate a gene library with appropriate activity [32]. CD14 was used as the screening substrate for specific screening. After competitive elution with LBP, we ultimately obtained 11 mutant clones that could compete with LBP for binding to CD14. Sequencing results showed that these 11 clones were located in the C-terminus of LBP, as was the peptide analog MP12 that was obtained in our previous studies. Nine of these 11 clones had T to M mutations at amino acid 287 of LBP, and the mutation rate was 81.8%.

We synthesized a polypeptide containing amino acids 252–291 of LBP according to the common sequence of these 9 clones and designated it P1. Among these, amino acid 287 was M and its amino acid sequence was FHRNHRSPVTLLAAVMSLPEEHNKMVYFAISDYVFNMASL. The length of the polypeptide containing the peptide analog MP12, which was previously screened out, still contained amino acids 252–291. Only amino acids 252–263 were substituted by MP12, and its amino acid sequence was FHRWPTWPLPSPAAVMSLPEEHNKMVYFAISDYVFNTASL. We then compared the in vivo and in vitro endotoxin activities of P1 and MP12.

LPS can induce most inflammatory cells to produce TNF-α [33],which is one of the main causes of ARDS. In addition to its direct toxic effects, TNF-α can interact with numerous molecules such as IL-1, IL-6 and IL-8 and cells, which results in more extensive damage to tissues [34]. The transcription factor NF-κB can induce the gene expressions for numerous inflammatory mediators, and inhibiting NF-κB activity can prevent the production of inflammatory mediators and reduce the damage to the lungs and other organs [35], [36].

In the current study we showed that mature U937 cells could express TNF-α and had induced NF-κB activity after LPS stimulation. When these cells were treated with LBP and LPS together, the extent of NF-κB activation and TNF-α expression were significantly increased. This suggested that LBP could bind to free LPS to form an LPS/LBP complex, which would deliver LBP to CD14 on the surfaces of monocytes and macrophages to amplify the inflammatory effects of LPS. However, LPS/LBP complex-induced NF-κB activation and TNF-α expression were significantly inhibited by both polypeptides P1 and MP12, and these induced effects were even lower than those with LPS treatment alone. These results showed that polypeptides P1 and MP12 could block the LBP site for binding to CD14, prevent LPS from binding to CD14 via LBP (LBP/LPS complex), interfere with the LBP/CD14 sensitizing system, and, thereby, inhibit LPS-induced release of inflammatory factors.

The polypeptides obtained from screening had an anti-inflammatory effect by blocking the LBP/CD14 binding site. This would not affect the LPS clearance and neutralizing effects of LBP. Thus, these polypeptides appeared to reduce the amounts of free LPS and inhibited LPS-induced inflammation. Moreover, these results showed that the inhibitory effect of polypeptide P1 on LPS-induced TNF-α expression and NF-κB activation was greater than that of MP12. A possible reason is that we established a mutant library for the C-terminus of LBP using epPCR and T7 phage display technology and the capacity of this library was up to 6.4×10^8^. This achieved the goal of screening out target proteins and optimizing function by affinity screening using CD14 as the substrate and competitive elution with LBP. Finally, we obtained an LBP/CD14 inhibitory peptide that had stronger activity for binding to CD14 and had a greater inhibiting effect on LPS-induced inflammation.

A T to M mutation occurred at amino acid 287 of LBP in 9 of 11 clones that were obtained by screening and this mutation rate was 81.8%. Polypeptide P1, which had this mutation site, exhibited significantly stronger biological activity for inhibiting LPS-induced inflammation. This result suggested that the site at amino acid 287 had an important role in LBP binding to CD14.

In our animal experiments, the arterial oxygen pressure and oxygenation index of rats decreased significantly after LPS injection, and there were pathological changes, including pulmonary edema, alveolar collapse, atelectasis, pulmonary hemorrhage, and neutrophil infiltration in rat lung tissues, which reflected ARDS. After treatment with P1 or MP12, the arterial oxygen pressure and oxygenation index improved and the lung tissue injury was reduced; the pathological scores were better than those of the LPS group. These experiments further demonstrated that LPS cold induce the release of inflammatory mediators and result in rat lung tissue injury.

Polypeptides P1 and MP12 probably blocked LBP binding to CD14 in rats, removed LPS due to the clearance effect of LBP, and thereby clear LPS from the circulation, which reduced the LPS-induced rat lung tissue injury.The improvements in rat arterial oxygen pressure, oxygenation index, and lung pathology scores were better in the P1 group compared to the MP12 group, which was in agreement with our in vitro experiments. This also suggested that the directed evolution of the C-terminus of LBP improved the effects of an LBP/CD14 inhibitory peptide on ameliorating LPS-induced inflammation.

In summary, we screened out the peptide analog MP12 in the LBP/CD14 binding site in our previous studies and localized it at amino acids 252–263 of the C-terminus of LBP. In the current study, we conducted directed evolution for this area using epPCR and T7 phage display technology to obtain polypeptide P1, which could block the LBP site for binding to CD14. Compare with MP12, the anti-endotoxin effects of P1 was probably slightly stronger than that of MP12, but they were weak. Thus, the further study of the comparison of anti-endotoxin activity between P1 and MP12 needs to be done later. In this study, 10 ug/ml of polypeptides was the only one dose adopted to compare the anti-endotoxin effects of P1 and MP12,and the best concentration of P1 need to study in our future work. In addition, we found that amino acid 287 may play an important role in LBP binding to CD14. This finding provided additional insights for realizing the structure and function of LBP, which would aid in developing a more ideal strategy for anti-endotoxin treatments.
